# Early gastric mixed neuroendocrine‐non‐neuroendocrine neoplasm with early poor prognosis after endoscopic submucosal dissection: A case report

**DOI:** 10.1002/deo2.10

**Published:** 2021-09-01

**Authors:** Yusuke Tomita, Hideyuki Seki, Emi Matsuzono, Yoshimitsu Kobayashi, Susumu Sogabe, Nozomu Sugai, Jun Fujita, Akira Suzuki

**Affiliations:** ^1^ Department of Gastroenterology KKR Sapporo Medical Center Hokkaido Japan; ^2^ Department of Pathology KKR Sapporo Medical Center Hokkaido Japan

**Keywords:** cancerization, early gastric cancer, ESD, MANEC, MiNENs

## Abstract

Early gastric mixed neuroendocrine‐non‐neuroendocrine neoplasms (MiNENs) are rare diseases, with no data on their incidence and prognosis. We report the case of intramucosal gastric MiNENs for endoscopic submucosal dissection (ESD) treatment. An 80‐year‐old male underwent esophagogastroduodenoscopy for screening and was suspected of early gastric cancer type 0‐IIa+IIc on the lesser curvature of the antrum, for which ESD treatment was performed. Histopathologically, the diagnosis was MiNENs. Synaptophysin‐positive adenoductal structures were observed in the adenocarcinoma component, suggesting that adenocarcinoma had dedifferentiated into neuroendocrine carcinoma. The tumor was located within the mucosal layer, with lympho‐vascular invasion. The patient was kept under observation; however, 6 months after the ESD, computed tomography scan revealed prominent ascites, enlarged lymph nodes, and liver metastases, and MiNENs were suspected to have poor prognosis. If MiNENs diagnosis is made preoperatively or postoperatively, surgical resection may be considered as treatment regardless of the tumor depth or lympho‐vascular invasion.

## INTRODUCTION

According to the 2010 World Health Organization classification, mixed adeno‐neuroendocrine carcinomas (MANECs) are tumors containing >30% adenocarcinoma and neuroendocrine carcinoma components. MANECs have been renamed as mixed neuroendocrine‐non‐neuroendocrine neoplasms (MiNENs) in the 2019 revised classification, in which neuroendocrine tumors are reclassified into benign neuroendocrine tumors and NECs.^1^ NEC is an infrequent diagnosis often detected in the advanced stage, and early gastric MiNENs are extremely rare,^2^, ^3^ wherein early gastric MiNEN can be defined as a gastric neoplasm that does not invade deeper than the submucosa. No study till date investigated the incidence and prognosis of early gastric MiNENs. We herein report the case of a patient with fast recurrence of MiNEN that was treated with endoscopic submucosal dissection (ESD); the patient was subsequently diagnosed with NEC arising from the adenocarcinoma component of the MiNEN.

## CASE REPORT

An 80‐year‐old male was admitted with suspicious early gastric cancer based on esophagogastroduodenoscopy screening. He had a history of ESD for early colorectal and prostate cancers treated with radiotherapy. His biochemical parameters and tumor markers were within normal limits.

**FIGURE 1:**
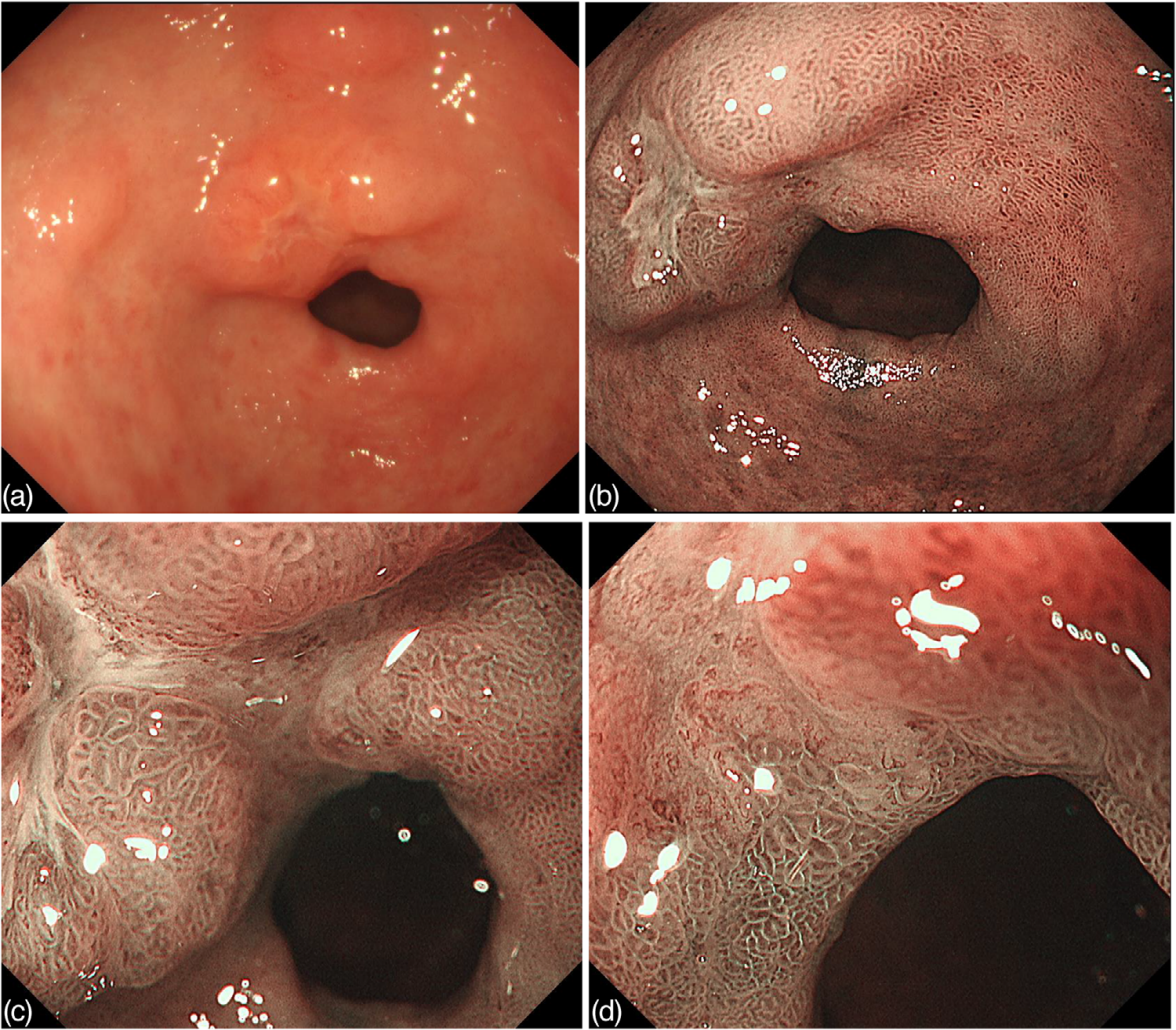
EGD findings. (A) An irregular depressed lesion with a protuberance area was found on the lesser curvature of the antrum. (B) Depressed lesion is divided into two parts. The oral depression had erosive changes and the surrounding elevated area showed SMT‐like elevation. The anal‐side depression was slightly flatter than the oral depression and had irregular margins and regional characteristics. Magnifying endoscopy with narrow‐band imaging. (C) In oral‐side depressed area, the surface structure had disappeared. (D) Anal‐side depressed area showed irregular microvascular architecture and microsurface structure with well‐demarcated line

Esophagogastroduodenoscopy showed no diffuse redness or enlargement of the folds of the background mucosa, but atrophic changes associated with previous H*. pylori* infection were observed. An irregular depressed lesion with a 20‐mm size protuberant area was found on the lesser curvature of the antrum (Figure 1a and b). The depressed lesion comprised two distinct areas: The oral side displayed erosive changes and the surrounding elevated area showed submucosal tumor‐like elevation. The depression on the anal side was slightly flatter than the oral‐side depression and had irregular margins and regionality. ME‐NBI revealed that most of the oral‐side depression surface was covered with white moss, which could not be observed in detail; however, some of the surface structures had disappeared (Figure 1c). The anal‐side depression exhibited irregular microvascular architecture and irregular microsurface structure with well‐demarcated lines (Figure 1d). Computed tomography (CT) scan revealed no lymph node or distant metastasis. Based on these findings, ESD was performed for 20‐mm type 0‐IIa+IIc lesion.

**FIGURE 2:**
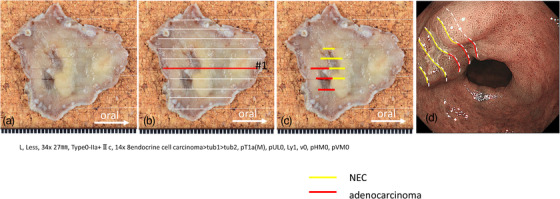
(A) The macroscopic finding of the specimens resected by endoscopic submucosal dissection. (B) Histopathological findings of the #1. (C) The pathological diagnosis was early gastric mixed neuroendocrine‐non‐neuroendocrine neoplasms (MiNENs). (D) The mapping indicates the relation between an endoscopic image and histology

**FIGURE 3:**
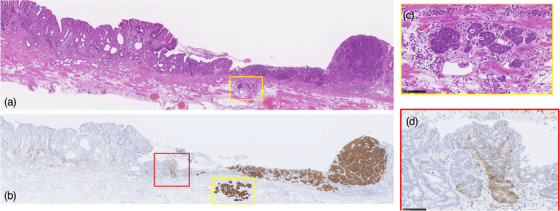
Loupe images of red line of the specimen. (A) Histopathological findings showed a well‐to‐moderately differentiated adenocarcinoma on the left side of the specimen and a substantial growth of tumor cells with granular chromatin, densely stained atypical nuclei, and small cytoplasm on the right side. The tumor shows no invasion into the submucosa. (B) Immunostaining showed that the tumor on the right side of the specimen was positive for synaptophysin and diagnosed as neuroendocrine cell carcinoma. The neuroendocrine cell carcinoma also showed lympho‐vascular invasion within the lamina muscularis mucosae. (C) The neuroendocrine cell carcinoma showed lympho‐vascular invasion within the lamina muscularis mucosae. (D) There were synaptophysin‐positive adenoductal formations in the adenocarcinoma component, suggesting that the adenocarcinoma had dedifferentiated into the neuroendocrine carcinoma (NEC)

The resected specimen had the following histologically parameters: L, Less, 34 × 27 mm, Type0‐IIa+Ⅱc, 14 × 8 endocrine cell carcinoma > tub1 > tub2, pT1a(M), pUL0, Ly1, v0, pHM0, pVM0 (Figure 2a). As shown in Figure 2b, histopathological examination revealed tub1/tub2 adenocarcinoma in the anal‐side depression. The oral‐side depression comprised proliferating tumor cells with hyperchromatic and dysmorphic nuclei (Figure 3a and b). The immunostaining was positive for synaptophysin for oral‐side depression, confirming the diagnosis of NEC. The Ki67 labeling index was high at approximately 90%. The neuroendocrine cell carcinoma also showed lympho‐vascular invasion within the lamina muscularis mucosae (Figure 3c). Furthermore, there were synaptophysin‐positive adenoductal structures in the adenocarcinoma component, suggesting that adenocarcinoma had dedifferentiated to NEC (Figure 3d). All tumors were confined to the mucosal layer.

The final diagnosis was early gastric MiNEN as the adenocarcinoma and NEC components independently accounted for >30% of the entire lesions. Figure 2c shows the adenocarcinoma (red lines) and NEC (yellow lines) components. The mapping indicates the relation between an endoscopic image and histology (Figure 2d).

Although the tumor was confined to the mucosa, additional surgical resection was planned due to the presence of lymphatic invasion. However, the patient declined treatment and was kept under observation. However, 6 months after ESD, he was admitted to the hospital with abdominal distention. CT scan revealed prominent ascites, enlarged lymph nodes, and liver metastases. Ascites cytology revealed synaptophysin‐positive cells, which led to the diagnosis of metastasis of NEC. The patient declined the recommended palliative chemotherapy and opted for best‐supported care. He died 20 days after the last CT scan.

## DISCUSSION

NEC comprises highly atypical neoplastic endocrine cells with high proliferative potential and rapid growth with poor prognosis, leading to metastasis from an early stage.^2^, ^3^ In the present case, half of the tumor was composed of adenocarcinoma, whereas the remaining half was NEC, leading to the diagnosis of MiNEN.

There are two hypotheses on MiNEN pathogenesis: (1) development of NEC due to dedifferentiation of adenocarcinoma cells during adenocarcinoma development, and (2) differentiation of epithelial stem cells, which have the capacity for multiple differentiations, into two components.^4^, ^5^, ^6^, ^7^, ^8^ The current case had discreet adenocarcinoma and NEC components in the mucosa and synaptophysin‐positive adenoductal structures in the adenocarcinoma component. This finding reflects a transition from adenocarcinoma to NEC, suggesting that NEC development might be due to the dedifferentiation of adenocarcinoma in the current case. This is the first study to show the transition from adenocarcinoma to NEC in the mucosa by pathologically capturing synaptophysin‐positive gland ducts and the first report of MiNEN.

Including the current case, only 14 cases of early gastric MiNEN, including MANEC, have been reported to date. Early gastric MiNEN was defined as a gastric neoplasm that does not invade deeper than the submucosa (Table 1), the prevalence of MiNENs was higher in males than in females, with males accounting for >80% of the cases; the incidence of MiNENs increased after the age of 60 years.

**TABLE 1:** Cases that reported early gastric MiNENs (including MANEC)

No.	Year	Author	Age	Sex	Size (mm)	Type	Depth	Component		Invasion	Treatment	Surface^*1^	Vascular^*2^	Preoperative biopsy	Clinical course
1	2013	Higuchi	80s	M	31 × 29	IIa	SM1	tub1‐muc	NEC	ly1v1	ESD	‐	‐	tub1	Recurrence in 2 weeks
2	2013	Lee	70	F	14 × 13	IIc	SM1	tub1	NEC	‐	ESD	‐	‐	tub1	No recurrence for 12 months
3	2014	Fukuba	80s	M	10 × 9	IIa	SM2	tub2	NEC	ly1v1	ESD surgery	‐	‐	tub2	No recurrence for 3 years
4	2015	Yamasaki	77	M	10 × 6	IIc	M	tub2	NEC	0	ESD	I	I	tub2	No recurrence for 7 months
5	2015	Yamauchi	76	M	22 × 22	IIc	SM	sig	NET	‐	surgery	‐	‐	por‐sig	‐
6	2016	Taguchi	63	M	23 × 20	IIc	SM2	tub2‐por	NEC	ly1v0	surgery	‐	‐	tub2	No recurrence for 16 months
7	2016	Sakatani	60s	F	8 × 7	IIc	SM1	tub12	NEC	‐	ESD surgery	A	I	tub2>tub1	No recurrence for 18 months
8	2017	Pastorello	61	M	20 × 15	‐	SM	tub‐por	NEC	ly1v0	surgery	‐	‐	NET	Recurrence in 6 years
9	2018	Kubo	80	M	25 × 22	IIa	M	tub1	NETG1	‐	ESD	‐	‐	‐	No recurrence for 6 months
10	2019	Jeong	66	M	20 × 8	IIa	SM?	tub2	NEC	‐	surgery	‐	‐	NET	‐
11	2019	Koseki	80s	M	9 × 8	IIc	SM2	tub1	NEC	ly0v1	ESD	A	I	tub2	No recurrence for 20 months
12	2019	Fujita	67	M	12 × 8	IIc	SM2	tub	NEC	ly1v0	ESD surgery	‐	‐	‐	No recurrence for 15 months
13	2020	Tanaka	80s	F	25 × 20	IIc	SM2	tub2	NEC	ly0v1	surgery	I	I	NEC	No recurrence for 6 months
		Our case	85	M	14 × 8	IIa+IIc	M	tub12	NEC	ly1v0	ESD	I/A	I	Group 4	Recurrence in 7 months

Abbreviations: A, absent; I, irregular; MANEC, mixed adeno‐neuroendocrine carcinomas; MiNENs, mixed neuroendocrine‐non‐neuroendocrine neoplasms.

^*^1: Surface pattern in ME‐NBI; ^*^2: vascular pattern in ME‐NBI.

Among the cases that showed the presence or absence of lympho‐vascular invasion, eight of the nine patients had lympho‐vascular invasion, suggesting that MiNEN involves high frequency of vascular invasion at an early stage. In addition, this was supported by our case in which there was lympho‐vascular invasion despite the presence of intramucosal carcinoma. In this cohort of 14 patients with early gastric MiNEN, ESD was performed in nine patients, including four and five patients who underwent curative and noncurative resection, respectively. For curative resection criteria for MiNENs in this report, we applied eCuraA and B according to the histology of the adenocarcinoma component of MiNENs in the guidelines established by the Japanese Gastric Cancer Association and Japan Gastroenterological Endoscopy Society. (The tumor diameter was defined as the size of both lesions). Surgical resection, including post‐ESD resection, was performed in eight patients; one of these eight patients experienced postoperative recurrence, whereas the remaining seven patients progressed without recurrence in the short term. Lymph node dissection may reduce the frequency of metastatic recurrence. In contrast, three patients underwent noncurative resection after ESD without additional surgery, and recurrence was observed in two patients, including the current case. Both patients had liver metastases and peritoneal dissemination during recurrence, which were early recurrences. Given that the criteria for endoscopic treatment of MiNEN remain unclear, it is necessary to explain the risk of recurrence in case follow‐up is provided.

A potential but unconfirmed early metastasis of MiNEN was reported by Ochiai et al.,^9^ who presented the case of a patient who initially underwent extended curative resection without vascular invasion for adenocarcinoma diagnosis for ESD treatment. The CT scan obtained 26 months later revealed lymph node metastasis, and positron emission tomography‐CT showed no other sites of overt accumulation except at the lymph node. Surgical resection was performed. In the postoperative pathological diagnosis, lymph node metastasis was diagnosed as MiNEN, and the reevaluation of the original ESD specimen showed partial CD56 positivity, although MiNEN was not diagnosed.

The present patient had intramucosal MiNEN with no obvious metastasis on preoperative CT but proceeded to a rapid outcome of death in 231 days from the initial diagnosis. The NEC component showed high Ki‐67 index (90%) and showed lympho‐vascular invasion in the ESD specimen. Therefore, the present case suggests that mixed tumors with an NEC component might have a strong tendency to invade and metastasize. ESD, a treatment option for MiNEN, is often not effective. Therefore, surgical resection should be considered when diagnosing MiNEN given the current lack of indications for endoscopic resection of neuroendocrine neoplasm. (To date, no indications exist for endoscopic resection of NEC. However, for NETs larger than 10 mm, surgical resection is indicated regardless of RINDI classification.). If NEC components are identified in ESD specimens of early‐stage gastric cancer, surgical resection should be performed with the patient's consent because of the risk of early recurrence as illustrated in the present case.

Unfortunately, we did not locate any reports of patients with a preoperative diagnosis of early gastric MiNEN. The reported ME‐NBI findings of early gastric NEC include loss of superficial structures and irregular vascular dilatation based on the comparison of preoperative endoscopic findings with resected specimens. Loss of superficial structures is often observed in poorly differentiated adenocarcinomas as well as in NEC. Therefore, it is possible that preoperative observation of NEC by ME‐NBI is mistakenly diagnosed as poorly differentiated adenocarcinoma due to the loss of superficial structures. In addition, NEC is rarely diagnosed by preoperative biopsy, which may be due to the similarity to poorly differentiated adenocarcinoma and the collection of insufficient biopsy material as NEC is primarily in the submucosa.^10^ Thus, early endoscopic diagnosis of MiNEN is difficult. In cases with preoperative findings of undifferentiated carcinoma, the possibility of MiNEN should be considered and postoperative pathological diagnosis should be performed to confirm the diagnosis of MiNEN.

In conclusion, we report the case of gastric MiNEN in the mucosa and summarize the available literature to emphasize that the incidence of lympho‐vascular invasion is high even in patients with early‐stage gastric MiNEN. Therefore, surgical resection rather than ESD should be considered for treatment in these patients. If NEC is suspected based on endoscopic findings, biopsy specimens should be obtained with rigorous immunohistochemical analysis before treatment, and detailed pathological examination of the resected specimen is required after treatment.

## CONFLICT OF INTEREST

The authors declare that there are no conflicts of interest.

## FUNDING INFORMATION

None.

## ETHICS APPROVAL

This study has been performed in accordance with the Declaration of Helsinki.
